# Differences in Trunk Accelerometry Between Frail and Nonfrail Elderly Persons in Sit-to-Stand and Stand-to-Sit Transitions Based on a Mobile Inertial Sensor

**DOI:** 10.2196/mhealth.2710

**Published:** 2013-08-16

**Authors:** Alejandro Galán-Mercant, Antonio I Cuesta-Vargas

**Affiliations:** ^1^Faculty of Health SciencesDepartment of PhysiotherapyUniversity of MalagaMalagaSpain; ^2^School of Clinical SciencesFaculty of HealthQueensland University of TechnologyBrisbaneAustralia

**Keywords:** frail syndrome, sit-to-stand, stand-to-sit, mobile phone, inertial sensor

## Abstract

**Background:**

Clinical frailty syndrome is a common geriatric syndrome, which is characterized by physiological reserve decreases and increased vulnerability. The changes associated to ageing and frailties are associated to changes in gait characteristics and the basic functional capacities. Traditional clinical evaluation of Sit-to-Stand (Si-St) and Stand-to-Sit (St-Si) transition is based on visual observation of joint angle motion to describe alterations in coordination and movement pattern. The latest generation smartphones often include inertial sensors with subunits such as accelerometers and gyroscopes, which can detect acceleration.

**Objective:**

Firstly, to describe the variability of the accelerations, angular velocity, and displacement of the trunk during the Sit-to-Stand and Stand-to-Sit transitions in two groups of frail and physically active elderly persons, through instrumentation with the iPhone 4 smartphone. Secondly, we want to analyze the differences between the two study groups.

**Methods:**

A cross-sectional study that involved 30 subjects over 65 years, 14 frail and 16 fit subjects. The participants were classified with frail syndrome by the Fried criteria. Linear acceleration was measured along three orthogonal axes using the iPhone 4 accelerometer. Each subject performed up to three successive Si-St and St-Si postural transitions using a standard chair with armrest.

**Results:**

Significant differences were found between the two groups of frail and fit elderly persons in the accelerometry and angular displacement variables obtained in the kinematic readings of the trunk during both transitions.

**Conclusions:**

The inertial sensor fitted in the iPhone 4 is able to study and analyze the kinematics of the Si-St and St-Si transitions in frail and physically active elderly persons. The accelerometry values for the frail elderly are lower than for the physically active elderly, while variability in the readings for the frail elderly is also lower than for the control group.

##  Introduction

Clinical frailty syndrome is a common geriatric syndrome which is characterized by physiological reserve decreases and increased vulnerability and which may, in the event of unexpected intercurrent processes, result in falls, hospitalization, institutionalization, or even death [1]. Detection and diagnosis of frailty depend on the following domains. *Medical:* Presence of chronic diseases, gait disturbance, sensory deficit, recurrent falls and hospitalization, and polypharmacy. *Functional:* Dependence in basic activities of daily living and instrumental activities of daily living. *Socioeconomic:* Living alone, recent widowhood aged over 80 years, and low income. *Cognitive and affective criteria:* Depression and cognitive impairment. *Need for institutionalization:* Live in retirement homes and nursing homes [2].

A previous study in the frailty detection [2] concludes that it is necessary to make a multimodal correct assessment of the whole clinical record. In this sense, this study established a set of clinical parameters from the laboratory report belonging to the patient record. Different groups of clinical factors had been identified: nutritional assessment, cognitive assessment, etc. The present study is focused only in the physical function. It however seems particularly worthwhile to measure mobility and balance (physical function) as a means of knowing whether acutely ill older people who are frail are recovering from their illness or becoming more ill [3-6].

The changes associated to ageing and frailties are associated to changes in gait characteristics and the basic functional capacities of the individual [7]. This variability in different movement patterns has been interpreted as a more conservative gait pattern in order to increase gait stability and reduce the risk of falls [8]. The new, more conservative gait pattern has greater cognitive involvement and produces a result focused entirely on movement, while the perception of unexpected trigger factors may be overlooked [9]. Dual tasks have been shown to affect normal gait development even in healthy persons [10].

As people get older, the ability to rise from a chair, usually labeled as the sit-to-stand (Si-St) and stand-to-sit (St-Si) postural transition, becomes a more demanding functional daily task [11,12]. Traditional clinical evaluation of Si-St and St-Si transition is based on visual observation of joint angle motion to describe alterations in coordination and movement pattern [11,12]. However, the validity of such assessment essentially depends on clinicians’ experience and training. Results might not have the precision needed to objectively assess the effect of rehabilitative intervention or the decline over time in frail elderly persons [11-13]. Kinematics of the trunk appears essential to maintain balance during Si-St transition [11,12,14]. The dynamic body movements using body-worn inertial sensors have been investigated [11,12,15-19].

A previous study concludes that inertial sensors can offer an accurate and reliable method to study human motion [20]. Algorithms for characterizing the gait of pedestrian using accelerometer and gyroscope signals recorded in a handheld device have been developed and presented in previous studies. This study concluded that the pattern recognition open new research options toward free-inertial tracking of pedestrian using handheld inertial sensors, which are already widely embedded in smartphones nowadays [21]. The latest generation smartphones often include inertial sensors with subunits such as accelerometers and gyroscopes that can detect acceleration and inclination [22]. The numerous applications developed for these smartphones mean the data from the accelerometer and the gyroscope can be read, stored, transferred, and displayed [23,24]. These applications evaluate and assess kinematic variables related to gait [25], measuring Cobb angles in x-rays, or as an objective method to classify levels of physical activity, and an indicator of the degree of functional capacity and quality of life [22,26].

There are two goals in the present study. First to describe the variability of the accelerations, angular velocity, and displacement of the trunk during the Si-St and St-Si transitions in two groups of frail and physically active elderly persons, through instrumentation with the iPhone 4 smartphone; and second, to analyze the differences and performance of the variance between the study groups (frail and healthy).

##  Methods

### Design and Participants

A cross-sectional study that involved 30 subjects over 65 years, 14 frail and 16 fit subjects. The participants were classified with frail syndrome by the Fried criteria (unintentional weight loss, self-reported exhaustion, weakness, slow walking speed, and low physical activity) [1]. Exclusion criteria were no history of pain in the last twelve months, previous surgery, presence of a tumor, and musculoskeletal disorders in the upper or lower extremity. Patients with impaired cognition, musculoskeletal back comorbidities, and problems associated with exercise intolerance were also excluded. A physiotherapist clinically examined all participants, and none of them have the exclusion criteria. Table 1 shows the characteristics of the sample.

Fit participants were recruited through advertisements in the Sport and Health Center in Torremolinos, Spain. Frail participants were recruited through advertisements in the Geriatrics Centers in Torremolinos and Benalmadena, Spain. Written informed consent was obtained from each individual. The ethics committee of the Faculty of Medicine at the University of Malaga, Spain approved the study.

### Data Collection and Procedures

Analysis was performed with SPSS version 15 for Windows while the data collection phase used inferential analysis between variables by type and normal. The nonparametric test Mann-Whitney [27] was used as determined by the variables normality of distribution. The statistical significance level was set at *P*<.05.

**Table 1:** Characteristics of sample (N=30).

	Mean		Standard deviation	
	Frail (n=14)	Fit (n=16)	Frail (n=14)	Fit (n=16)
Age (years)	83.71	70.25	6.37	3.32
Weight (kg)^a^	56.21	71.03	9.64	13.11
Height (cm)^b^	155.79	159.44	7.81	10.61
Body mass index (kg/m^2^)^c^	23.36	27.87	3.48	3.79

^a^kg=kilograms

^b^cm=centimeters

^c^m=meters

## Results

Table 2 summarizes the acceleration-based measures of the Si-St and St-Si transitions in the two groups.

Table 3 summarizes the gyroscope-based measures of the Si-St and St-Si transitions in the two groups.

**Table 2:** Acceleration-based values from the Si-St and St-Si transitions (N=30).

			Mean			Standard deviation					
			Frail (n=14)		Fit (n=16)	Frail (n=14)		Fit (n=16)	U^a^		*P* value
**Si-St** (m/s^2^)^b,c^											
	*x* ^d^.acc^e^.max^f^	2.004			3.353	0.761		1.475	49.50		.009
	*x*.acc.min^g^	-1.443			-3.136	1.211		1.198	30.50		<.001
	*y* ^h^.acc.max	3.069			6.248	1.240		1.913	15.00		<.001
	*y*.acc.min	-1.471			-6.182	0.788		2.415	0.000		<.001
	*y*.acc.mean	0.668			0.018	0.513		0.690	45.00		.005
	RV^i^.acc.max	7.065			8.972	2.233		2.506	58.00		.025
	RV.acc.mean	2.975			4.215	1.063		0.964	44.00		.005
**St-Si** (m/s^2^).											
	*y*.acc.max	3.567			6.200	2.028		1.752	26.50		<.001
	*y*.acc.min	-2.950			-9.003	2.441		4.334	14.00		<.001
	*z* ^j^.acc.max	5.830			3.834	2.170		2.682	62.00		.038
	*z*.acc.min	-3.770			-6.645	1.928		2.374	35.00		<.001
	*z*.acc.mean	0.874			-1.611	1.672		1.701	36.00		.002
	RV.acc.max	7.213			10.652	2.566		3.501	41.00		.003
	RV.acc.min	0.364			0.808	0.255		0.479	38.00		.002
	RV.acc.mean	3.188		4.263			0.708	1.048	45.00	.005	

^a^U=U-Mann-Whitney

^b^m=meters

^c^s=second

^d^ *x*=*x*-axis

^e^acc=acceleration

^f^max=maximum

^g^min=minimum

^h^ *y=y*-axis

^i^RV=resultant vector

^j^ *z=z*-axis

**Table 3:** Gyroscope-based values from the Si-St and St-Si transitions (N=30).

					Mean				Standard deviation				
					Frail (n=14)		Fit (n=16)		Frail (n=14)	Fit (n=16)	U^a^	*P* value	
**Si-St**													
		roll.rotation.max^b^(deg)^c^		102.920		196.544		98.755		109.519	35.00		.001
		roll.rotation.mean (deg)		-24.754		83.837		58.165		150.560	61.00		.034
		rate^d^.yaw.min^e^(deg/s)^f^		-47.813		-26.131		17.501		25.998	42.00		.004
		rate.pitch.max (deg/s)		27.414		123.404		15.552		141.318	28.00		<.001
		rate.roll.max (deg/s)		18.924		165.437		8.843		120.989	0.000		<.001
		rate.roll.min (deg/s)		-19.796		-62.597		10.956		39.321	56.00		.020
		rate.roll.mean (deg/s)		0.459		49.993		1.289		82.129	59,00		.028
**St-Si**													
	roll.rotation.min (deg)		-163.264			-61.157		38.955		108.358	60.00		.031
	roll.rotation.mean (deg)		-15.487			83.102		40.876		142.182	62.00		.038
	rate.yaw.max (deg/s)		41.309			130.470		11.316		138.379	57.00		.022
	rate.yaw.min (deg/s)		-67.449			-37.077		21.053		30.776	49.00		.009
	rate.roll.max (deg/s)		38.146			145.150		18.918		129.161	13.00		<.001
	rate.roll.min (deg/s)		-25.596			-70.275		16.433		50.714	58.00		.025

^a^U=U-Mann-Whitney

^b^max=maximum

^c^deg=degrees

^d^rate=angular velocity

^e^min=minimum

^f^s=second

##  Discussion

### Principal Results

The present study has described and examined the identification, analysis, and differentiation in the performance of kinematic variables using the inertial sensor fitted in the iPhone 4 during Si-St and St-Si transitions in healthy and frail elderly people. Significant differences were found between the groups of elderly people in the accelerometry and angular displacement variables obtained in the kinematic readings of the trunk during the both transitions.

The results obtained in this study show a series of weakness in the frail elderly population group. The most significant deficits found in the Si-St and St-Si transitions corresponded to accelerometry (see Table 2), with the frail elderly persons obtaining lower maximum and minimum accelerations than the physically active elderly persons in the y-axis during these transitions.

### Comparison With Prior Work

As far as we are aware, this is the first study that has used iPhone 4 technology to analyze and study the kinematics of healthy and frail persons aged over 65 years during the Si-St and St-Si transitions. Moreover, it is the first study that has shown the possibility of differentiating kinematic patterns in both transitions. The instrumented kinematic analysis of the Si-St and St-Si transitions was analyzed previously [11,15-19]. Moreover, in these studies no data were provided regarding magnitudes of acceleration and angular velocity obtained with a smartphone. By way of example, the results of the present study obtained in Table 2 show kinematic data that inform us that in the Si-St transition the linear acceleration of the trunk on the y-axis showed significant differences between healthy and frail elderly persons, while linear acceleration in the z-axis did not show any statistically significant differences.

It should be noted that frailty is defined as a clinical syndrome in which three or more of the following criteria should be present: unintentional weight loss, self-referred exhaustion, muscular weakness, low walking speed, and low physical activity levels [1]. Generically, the gyroscope and accelerometry data obtained for the Si-St and St-Si transitions were similar to other studies with other types of study groups or other types of functional tasks. In the present study, the frail elderly showed low magnitudes in the kinematic values with low variability (very small standard deviations) compared to the controls, the same as the subjects affected by Parkinson's disease [28-30], the elderly with a high risk of falls [7] and the frail elderly in a previous study [11,12,31].

There are two recent studies [11,12] that have instrumented the Si-St and St-Si transitions, differentiating and analyzing the kinematic data in each of the transitions between two groups of elderly persons. However, unlike the present study, they did not use iPhone 4 technology to collect kinematic variables.

Another recent study which has worked on the instrumentalization of the Timed Get Up and Go [7] test systematically evaluated the accelerometry values in elderly persons with a high risk of falls during the traditional three meter test, focusing solely on transitions in Si-St and St-Si. Like the present study, this study found numerous variables deriving from acceleration that showed differences between groups. However, the variables in this study were different, as was the methodology, and other things. Moreover, the measurement units were not coincident, and this study was based on the acceleration increase amplitude and the acceleration slope.

From a clinical perspective, the present study demonstrates that these new accelerometry parameters play an important role in differentiating between subjects with different functional states. These results provide new knowledge, extending existing knowledge on the isolated study of Si-St and St-Si transitions in frail and physically active elderly persons [11,12,31].

With regards to analysis of the data obtained in the present study, the differences between the frail and the physically active elderly show a series of deficits in the group of frail persons in both transitions. It is notable that the most significant deficit for the frail elderly in the Si-St and St-Si corresponded to accelerometry, with the frail elderly obtaining much lower minimum and maximum accelerations than the physically active elderly in the y-axis (see Table 2). In kinematic terms, this axis corresponds to accelerations in the VT-axis, leading us to believe that the frail elderly have less strength to carry out the impulse in concentric contraction of the quadriceps femoris muscle and the decrease in eccentric contraction of the same muscle on the VT-axis, as required for the transition from sitting to standing and vice versa. A study of the factors which influence this transition in 669 institutionalized elderly people showed that quadricep strength is the most important determinant factor for this transition, although there are other factors such as proprioception, movement execution speed, and psychological aspects which also influence ability to successfully carry out this functional test. Other factors, which may influence this transition, are foot position, anthropometry of the individual, or the height of the chair [16].

### Limitations and Future Work

The present findings motivate future investigations along these lines, but this study presents some limitations. First, men and women have different characteristics and it would be very interesting to provide the differences between them after the St-Si/Si-St exercise. A new study is needed to compare between genders. Moreover, prospective studies are needed to determine if acceleration-derived measures, perhaps in combination with other metrics and previously described measures of fall risk, can predict. Additional work is also needed to explore other properties of accelerometer-derived measures of the Si-St/St-Si. Note that here we specifically focused on timing of transition and a subset of the properties of the signal; further analysis of the complete waveform and other time points may provide additional utility. Indeed, it appears that the proposed approach not only may offer a more refined scale for assessing older adults, but it may also help to pinpoint specific problems that give rise to an abnormal performance of functional tasks (eg, Si-St/St-Si transitions). In the meantime, the present results demonstrate the potential of using an accelerometer to measure Si-St/St-Si performance, while maintaining simplicity and requiring no additional time to acquire the data.

### Conclusions

The inertial sensor fitted in the iPhone 4 is able to study and analyze the kinematics of the Si-St and St-Si transitions in frail and physically active elderly persons. The accelerometry values for the frail elderly are lower than for the physically active elderly, while variability in the readings for the frail elderly is also lower than for the control group. This suggests that the frail elderly carry out the test in a more careful, restricted way during the functional tasks, which make up the transitions, possibly showing their reduced ability to regulate movement when performing these tasks and transitions. The compensation mechanisms also play an important role. These results indicate that the additional, relevant information for future discriminant analysis comes mainly from the acceleration signal during the Si-St and St-Si transitions.

## Abbreviations

RPY: roll, pitch, and yaw
RV: resultant vector
SDs: standard deviations
SI-ST: sit-to-stand
ST-SI: stand-to-sit
VT: vertical axis
